# What do we mean by “lessons learned”? Medical didactic research deficits before the post-COVID era. A call!

**DOI:** 10.3205/zma001521

**Published:** 2021-11-15

**Authors:** Christoph Stosch, Kai P. Schnabel

**Affiliations:** 1 University of Cologne, Faculty of Medicine, Office of the Dean of Studies, Cologne, Germany; 2 University Bern, Institute for Medical Education, AUM, Bern, Switzerland

## Editorial

Attempting to classify the articles published on medical didactics in times of the COVID-19 pandemic in the JME since the beginning of the pandemic (reference to the COVID-19 pandemic in the title (years 2020 and 2021) or included in the two special issues 07/2020 and 01/2021), we find 84 articles with a brief search. Five of them are editorials, 56 articles deal with the digitization of teaching (lectures and seminars, courses and skills training, simulation persons or real patients, continuing education, selection procedures, etc.), 15 with curricular changes or modified teaching organization (support of digitization by auxiliary staff, for example) and 8 articles with other topics. The articles are mostly “best practice examples”, mostly only with acceptance studies. Only 5 articles of these deal further with the effects of the teaching change in the COVID-19 pandemic on the education, the outcome of the students [1], [2], [3], [4], [5]. 

At the same time, we may all have our fears, gut feeling or even quiet suspicion that the forced digitalization of wide parts of studies cannot be without effect on (self-)education resp. professional transformation in the health professions: “Most lecturers would like to teach more digitally even after the pandemic but fear a decrease in learning effectiveness and contact with students (...).” [6] concludes Speidel et al. in the latest, digital issue of JME, for example. Or do these fears merely reveal a new variety of structurally conservative critique of change that groundlessly clings to the known, whatever that may be, or more unpolemically, however the evidence-backed status quo might be described in terms of studying? To argue otherwise: Suppose we found no demonstrable changes in learning behaviors, knowledge stocks, physician attitudes, or skills and competency practice. Would it then be permissible to call the (digital) substitutes obviously sufficient and move on to business as usual? Or would this make us the gravediggers of patient-centered teaching, which is elaborately orchestrated everywhere but is ineffective? 

This could be exactly the case, according to Haase-Fielitz et al. in this issue of JME [7], who give practical teaching of resuscitation skills – albeit in a monocentric study – a poor report. Knowledge, attitudes and behavior regarding vaccination medicine in medical trainees in health care professions also suggest room for improvement [8] while Kruse et al. [9] and Schlegel et al. [10] highlight with “Deaf awareness” and “Onomatopoeia” two topics which are not or not sufficiently taught in the teaching of communication skills in the view of the authors. 

While Boehme et al. [11] describe the preparation and the – not unproblematic – implementation status of digitalization in a nationwide survey, Simmenroth et al. [12] present a concrete, semi-digital teaching scenario on “Alcohol and Smoking Counseling”. López Dávila et al. [13] describe nationwide quality care in the recognition of medical degrees earned abroad in Costa Rica, and Pentzek et al. [14] examine quality development of general medical clerkships through collegial feedback. Nikendei et al. describe [15] compensatory effects of voluntary assignments to support COVID-19 patients by students on, for example, “professional identification” while bedside teaching was absent, and Rohr et al. describe positive attitudes toward optional teaching components, in this case visionary elective curricula, in their article [16].

So the question now, and in particular with reference to the last two articles, is: Will our students become good healthcare workers because of or despite our curriculum? Investigating this in light of the changed curricula around the COVID pandemic as a large-scale digital experiment, with all the limitations of retrospective cohort studies and other methods, seems the order of the day. Valid, objective, and reliable measures of outcomes are also urgently needed, even if, as we all know, this was not possible during the pandemic. Now we should take the time to measure outcomes with appropriate methods that go beyond a mere satisfaction measurement of the participants – without wanting to minimize this as a necessary prerequisite of good teaching (!). How can we not throw the baby out with the bathwater in the transitional phase amidst the strong desire to return to face-to-face instruction and adequately examine online formats introduced in the pandemic with face-to-face formats? How can we increase efficiency while maintaining effectiveness? Can we initiate randomized crossover studies within cohorts and offer parts online or face-to-face? Solid education research is needed here and more necessary than ever! 

## Competing interests

The authors declare that they have no competing interests. 
